# Resilience and Social–Emotional Expertise as Predictors of Problematic Internet Use Among University Students

**DOI:** 10.3390/bs15050650

**Published:** 2025-05-10

**Authors:** Gözde Önal, Turan Emre Özdemir

**Affiliations:** Department of Occupational Therapy, Faculty of Health Sciences, Ankara Medipol University, Ankara 06050, Turkey; turan.ozdemir@ankaramedipol.edu.tr

**Keywords:** resilience, problematic internet use, social–emotional expertise, university student, predictor

## Abstract

Problematic internet use has become an increasing concern among university students, as it may negatively affect academic performance, emotional well-being, and social functioning. Understanding the psychological and emotional factors that influence internet use is crucial to developing effective preventive strategies. This study aimed to examine the relationship between resilience and social–emotional competence and problematic internet use among university students. This study was conducted with the participation of 191 students. The students’ problematic internet use levels were assessed using the Generalized Problematic Internet Use Scale-2, their resilience levels were assessed using the Connor–Davidson Resilience Scale, and their social–emotional competence levels were assessed using the Social–Emotional Competence Scale. Regression analysis was performed using the elastic net regression model and partial least squares (PLC) model. The general resilience level (*p* = 0.0015) and its sub-dimensions of tenacity (*p* = 0.0014), tolerance to negative affect (*p* = 0.0114), and spirituality (*p* = 0.0278) were found to be significant predictors of problematic internet use. The general social emotional competence level (*p* = 0.0115) and adaptability (*p* = 0.0278) were found to significantly predict problematic internet use. The predictive factors for the social interaction domain of problematic internet use were tenacity (*p* = 0.04), adaptability (*p* = 0.02), and expressivity (*p* = 0.03), while for negative results, they were tolerance to negative events (*p* = 0.05), spirituality (*p* = 0.04), and adaptability (*p* = 0.05). The factors affecting emotional regulation were tenacity (*p* = 0.03), spirituality (*p* = 0.03), adaptability (*p* = 0.03), and expressivity (*p* = 0.03). Only the spirituality (*p* = 0.05) and expressivity (*p* = 0.04) levels predicted insufficient self-regulation. The effects of the resilience and social–emotional competence levels on problematic internet use should not be ignored. In the plans and interventions to be developed, it is of great importance to take measures to improve the level of resilience and social–emotional competence skills.

## 1. Introduction

The internet is known as a global network that connects devices around the world and has become an indispensable part of daily life. With the development of technology in recent years, it has become very easy for individuals to access the internet with devices such as smartphones, tablets, computers, etc. It is known that 5.35 billion people, or approximately 68% of the world’s population, have access to the internet today (Social, 2024). According to the data of the Turkish Statistical Institute (2023), the internet usage rate of individuals between the ages of 16 and 74 in Turkey is reported to be 87.1% (Turkish Statistical Institute, 2023). When the internet activities of users are examined, it is seen that there is intensive use in different areas, such as social media, online video platforms, e-mail usage, online shopping, etc. (Social, 2024). Although the internet makes human life easier with its unique opportunities, excessive and uncontrolled use may lead to problematic internet use, which has been associated with depression, social isolation, obesity, sleep disorders, and attention deficit (Anderson et al., 2017; Reed & Reay, 2015; Cai et al., 2023).

Problematic internet use refers to internet use that negatively affects individuals’ work and social lives but has not reached the level of addiction (Caplan, 2002). To plan the necessary interventions to reduce problematic internet use before it reaches the level of addiction, it is important to examine the factors affecting problematic internet use. University students are considered a high-risk group for problematic internet use (Anderson et al., 2017). This increased vulnerability is primarily associated with factors such as difficulties with time management, unsupervised and unlimited internet access, and not yet having a fully developed self-control capacity, which is characteristic of emerging adulthood (Kuss & Griffiths, 2012). On the other hand, problematic internet use can result in adverse outcomes, including reduced academic performance, social withdrawal, and psychological difficulties (D. Li et al., 2010; Jun & Choi, 2015). These consequences may, in turn, reinforce problematic use. This tendency is particularly evident in individuals who experience difficulties in face-to-face social interactions, as they may turn to online engagement as a coping strategy. Such behavior can further exacerbate social isolation and ultimately create a vicious cycle (Kardefelt-Winther, 2014; Young, 2004). Problematic internet use has been linked to effects on students’ academic performance resulting in lower grades and potential academic setbacks (Jun & Choi, 2015). Furthermore, excessive internet use can have effects on students’ physical well-being, contributing to health issues like obesity and sleep disturbances (Anderson et al., 2017).

Understanding and expressing emotions when building and nurturing relationships are key components of social–emotional expertise according to (Goleman, 1996). This expertise encompasses a range of abilities, including empathy, accountability, decision making and awareness of dynamics, as noted by Fitzgerald et al. (2022). Recently, it has been accepted that social–emotional expertise has an important place in childhood, adolescence, and adulthood (C. Chen et al., 2021). Especially, university students face various academic and social difficulties as they enter a new environment (Akhtar & Akhtar, 2024). Academic knowledge and skills alone are not sufficient to cope with challenges, manage stress, and establish healthy relationships. It is known that students with developed social–emotional skills are more successful academically and socially, as well as stronger psychologically and more resistant to stress (Fitzgerald et al., 2022; Z. Li et al., 2024; Masten, 2021).

Resilience is defined as the ability to show flexibility, maintain a positive outlook, and adapt in the face of stress and challenges (Snyder & Lopez, 2001). It is known that the factors affecting resilience in university students include family and peer relationships, optimism, psychological well-being, physical activity, and academic life (Auttama et al., 2021; Thomas & Revell, 2016; Zaheer & Khan, 2022). Factors such as depression, anxiety, and stress that affect the resilience of university students are significantly related to problematic internet use, so problematic internet use is an issue that should be taken into consideration (Abdelgany et al., 2018; Akhter & Khalek, 2020; Reed & Reay, 2015).

Factors such as problematic internet use, social–emotional expertise, and resilience have been examined in the literature (Chong et al., 2014; Gioia et al., 2021; Olenik Shemesh et al., 2024). Chong et al. (2014) discovered a link between internet use and reduced resilience in college students, leading to feelings of depression. Gioia et al. (2021) highlighted the relationship between resilience and excessive internet use, noting its impact on skills. Olenik Shemesh et al. (2024) explored how social–emotional expertise relates to internet use, indicating that students with emotional skills are less likely to engage in excessive online behavior.

Previous studies have mostly examined resilience and social–emotional expertise separately as protective factors against problematic internet use (Chong et al., 2014; Gioia et al., 2021; Olenik Shemesh et al., 2024). However, these constructs represent distinct yet complementary domains of psychological functioning. While resilience reflects an individual’s capacity to cope with adversity and internal distress (Smith et al., 2008), social–emotional expertise involves emotional awareness, self-regulation, and interpersonal communication, which shape how one navigates social demands (Zhou & Ee, 2012). Investigating these variables simultaneously provides a more holistic understanding of the psychosocial resources that protect university students who are navigating a period of emerging adulthood marked by emotional, academic, and relational challenges (Arnett, 2000). Therefore, the present study offers a novel contribution by examining both the individual and combined effects of resilience and social–emotional expertise in predicting problematic internet use. The findings may inform the development of preventive interventions targeting both intrapersonal and interpersonal skills to mitigate internet-related risks in young adults.

## 2. Materials and Methods

### 2.1. Study Design

This study is a descriptive cross-sectional study that aims to investigate the effects of resilience and social–emotional expertise on problematic internet use among university students.

### 2.2. Sample Selection

The mean age of the students participating in this study was calculated as 21.6 ± 2.74 years (minimum 18, maximum 42). There were 148 (77.5%) female and 43 (22.5%) male students in this study. All the students were actively enrolled in associate or undergraduate programs from a variety of academic fields, including health sciences, social sciences, education, and engineering.

The required sample size was calculated using R software (v4.3.3), based on a 95% confidence level and 90% statistical power, which indicated a minimum of 178 participants. To meet this requirement, this study was introduced to students during classroom sessions at a university in Ankara. Approximately 400 students were informed about this study and invited to participate. Among them, 235 students voluntarily accessed the Google Forms link and consented to participate. Of these, 44 students were excluded due to self-reported health conditions (e.g., anxiety, attention deficit hyperactivity disorder), resulting in a final sample of 191 participants. The inclusion criteria were as follows: (1) being actively enrolled in an associate or undergraduate program, and (2) having the ability to read and understand Turkish. Individuals with known cognitive, psychiatric, or physical disabilities were excluded from this study. These were assessed through direct items on the sociodemographic information form. A convenience sampling method was employed based on accessibility and voluntary participation. Participation was voluntary and anonymous, and no personally identifying data were collected. All the participants provided their informed consent digitally prior to participation, and no compensation or incentive was provided for their involvement in this study.

### 2.3. Procedure

Participants were selected from a university in Ankara. The research invitation was shared via in-class announcements and communication with academic staff and student groups across different departments. Additionally, faculty members introduced this study during relevant course sessions. Students who were interested accessed the Google-Forms-based questionnaire, which included the informed consent form, sociodemographic form, and assessment tools.

This study was conducted through face-to-face sessions in classroom settings, where students completed the questionnaire using their personal devices (e.g., smartphones). Prior to participation, students were provided with standardized information regarding the purpose, voluntary nature, and ethical principles of this study. They were also informed that they could withdraw at any time without any consequences. Digital informed consent was obtained from all the participants before they could proceed with the survey. No personal identifying data were collected, and anonymity was fully ensured.

The average time taken to complete the survey was approximately 15–20 min. The assessment tools included the Generalized Problematic Internet Use Scale-2 (GPIUS-2) to measure problematic internet use, the Connor–Davidson Resilience Scale (CD-RISC) to evaluate resilience, and the Social–Emotional Expertise Scale (SEE) to assess social–emotional expertise. Our study was approved by the non-interventional clinical research ethics committee of a university.

### 2.4. Measurements

Sociodemographic Form: Contains questions that evaluate the participants’ age, gender, department, grade level, daily sleep duration and internet usage purposes, etc.

Generalized Problematic Internet Use Scale-2 (GPIUS 2): The instrument was developed by Caplan to determine the tendency toward problematic internet use (Caplan, 2010). The scale consists of a total of 15 items and four sub-dimensions rated on an 8-point Likert-type scale (1 = Strongly Disagree, 8 = Strongly Agree). The sub-dimensions of the scale are emotion regulation, insufficient self-regulation, social interaction and negative results. Emotion regulation refers to the use of the internet to cope with or escape from negative emotions. Insufficient self-regulation reflects a combination of compulsive use and cognitive preoccupation, indicating difficulties in controlling internet use and persistent mental engagement with online content. Social interaction assesses the preference for online communication over face-to-face interaction. Negative results captures the extent to which internet use leads to adverse personal, academic, or social consequences (Ayazseven & Önder, 2019).

The scale gives a general score regarding the tendency toward internet use and describes problematic internet use in each sub-dimension. The internal consistency coefficient of the original scale is 0.91. The adaptation of the scale to Turkish was conducted by Ayazseven and Önder, and the Cronbach’s alpha reliability coefficient of the Turkish version of the Generalized Problematic Internet Use-2 was found to be 0.85 (Ayazseven & Önder, 2019). According to the confirmatory factor analysis results of the original scale, the “Compulsive Internet Use” and “Cognitive Preoccupation” sub-dimensions in the Turkish version of the scale were collected under the “Insufficient Self-Regulation” sub-dimension. The lowest score that can be obtained from the scale is 15, and the highest score is 120. Increasing scores from the scale indicate an increasing tendency toward problematic internet use (Ayazseven & Önder, 2019). In this study, the Cronbach’s alpha coefficient for the total scale was 0.63.

Connor–Davidson Resilience Scale (CD-RICS): The instrument was developed by Connor and Davidson to measure the resilience levels of individuals (Connor & Davidson, 2003). The Turkish validity and reliability study of the scale was conducted by Karaırmak (Karaırmak, 2010). The scale consists of 25 questions in total, and the answers are scored on a 5-point Likert-type scale, with each item scored between 0 and 4 points: never true (0 points) and almost always true (4 points). The lowest score that can be obtained from the scale is 0, and the highest score is 100. A high score indicates high resilience. The scale has 3 sub-dimensions: tenacity and personal expertise, which reflects determination and belief in one’s own competence; tolerance to negative affect, which refers to the ability to endure distress without becoming overwhelmed; and spirituality, which captures faith-based coping strategies.

The Cronbach’s alpha coefficient of the Turkish version of the scale was obtained as 0.92, while the Cronbach’s alpha coefficients of the tenacity and personal expertise, tolerance to negative affect and spirituality sub-dimensions were obtained as 0.93 (15 items), 0.79 (6 items) and 0.50 (3 items), respectively (Karaırmak, 2010). In the current study, the Cronbach’s alpha coefficient for the full scale was 0.62.

Social–Emotional Expertise (SEE) Scale: The instrument was developed by McBrien et al. to measure the concept of social–emotional expertise (McBrien et al., 2020). The Turkish adaptation, validity and reliability study of the scale was conducted by Ay and Temel (2021). The scale, consisting of 25 questions, is a 5-point Likert-type scale. Each question is answered between “I completely disagree” 1 point and “I completely agree” 5 points. The lowest score that can be obtained from the scale is 25 and the highest score is 100. A high score on the scale indicates a high level of social and emotional expertise. The scale consists of 2 sub-dimensions: adaptability, which assesses the ability to adjust one’s behavior across different social situations, and expressivity, which measures the extent to which individuals comfortably express emotions in social interactions.

For the Turkish version of the scale, the Cronbach’s alpha values of the sub-dimensions were calculated as 0.804 for the adaptability sub-dimension and 0.734 for the expressivity sub-dimension (Ay & Temel, 2021). In the current study, the Cronbach’s alpha was 0.79 for the total scale.

### 2.5. Data Analysis

Statistical analyses were conducted using SPSS (version 25) and Python (version 3.8). The variables were investigated using visual (histograms and probability plots) and analytical methods (Kolmogorov–Smirnov/Shapiro–Wilk’s test) to determine whether they are normally distributed. The mean, standard deviation and minimum–maximum values were calculated for the numerical variables; frequency tables were created for the ordinal variables.

While creating the regression models, the independent variables were determined as the resilience levels and social–emotional expertise levels of university students, and the dependent variables were determined as problematic internet use levels. The relationship between the dependent and independent variables was analyzed with the Pearson correlation test.

It was planned to create two regression models. In the first model, it was aimed to determine the general effect of resilience and its sub-dimensions, as well as social–emotional expertise skills and its sub-dimensions, on problematic internet use. In the second model, it was aimed to determine in detail the effects of the resilience sub-dimensions and social–emotional expertise sub-dimensions on the problematic internet use sub-dimensions.

Before deciding on the appropriate regression model, the assumptions were checked as follows: (1) all the dependent and independent variables were a continuous data type, (2) the dependent variable (or better, the residuals) were normally distributed, (3) there were no outliers in the data, (4) each independent variable, when considered individually, did not show a correlation of more than 0.30 with the dependent variable, which met the assumption of the linearity of correlation, and (5) when the independent variables were considered pairwise, it was seen that some variables showed a correlation of more than 0.70 with each other and the assumption of multicollinearity was violated. In addition, the variance inflation factor (VIF) values, which were required to be below 10 for multicollinearity, were calculated to be above 10 for some variables, while (6) the residuals vs. predicted values plot showed that the residuals were randomly distributed, and the assumption of homoscedasticity was also met. When the regression analysis assumptions were evaluated, it was determined that there was multicollinearity and overfitting among the independent variables. For this reason, in the first model, the elastic net regression model was used instead of the multiple linear regression model, and in the second model, the partial least squares (PLS) regression model was used instead of the multivariate regression model.

In the case of multicollinearity, the estimated coefficients may be very unstable and therefore far from their target values. Thus, the estimates made by the regression model may be very weak (Polat & Günay, 2009). The elastic net regression model is defined as a good method to deal with highly correlated groups of estimates, which is used to estimate the regular linear regression models for a dependent variable on one or more independent variables (Aytekin, 2021). It provides the benefits of both lasso and ridge regression. It provides regularization through a ridge-type penalty and feature selection through a lasso-like penalty (Zou & Hastie, 2005). Lasso regression, which has better predictive power, is used while performing feature selection. It combines the best aspects of both models in a single model by incorporating both the feature group selection of ridge regression and the feature selection of lasso regression (Aytekin, 2021). Therefore, the elastic net regression model was used instead of the multiple linear regression model in the first model. The coefficients and cross-validation scores were used while creating the elastic net regression model. While the coefficients show the effects of the independent variables on problematic internet use, the cross-validation score measures the performance and accuracy of the model. The bootstrap method was used to evaluate the coefficients and *p*-values of the elastic net regression model. In the bootstrap method, samples were taken repeatedly (1000 times) from the original dataset. Each sample was set to include 80% of the original dataset. The elastic net regression model was established for each sample and the coefficients of the independent variables were calculated. The mean and standard error of the coefficients calculated for all the samples were calculated. The Z-scores were calculated using the mean and standard error of the coefficients, and the Z-scores were converted to *p*-values to evaluate the significance of the coefficients.

In the second model, the PLS regression model was used instead of the multivariate regression model. PLS regression can be considered an extension of linear regression. It is based on the idea of reducing variables to fewer components that do not have multicollinearity problems and establishing a regression model. PLS finds the components that have the highest correlation with both the independent variables and the dependent variable. In other words, it is used to best explain the structure between independent variables with both the dependent variable and the independent variables. The primary objective of PLS is to address regression issues, essentially focusing on forecasting the variable. PLS aims to predict or analyze a group of variables based on a set of variables. To accomplish this prediction, a distinct set of components possessing the capabilities is derived from the independent variables. These distinctive components, known as variables, according to Abdi (2003), are orthogonal in nature. Particularly beneficial for scenarios involving variables, the PLS approach introduces novel independent factors or latent variables to establish the relationships between dependent and independent variables. Each component represents a combination of variables with subsequent utilization of standard regression techniques to establish the connections between these derived components and the dependent variables. Within the scope of this research, the bootstrap method was used to evaluate the reliability of the model and the significance of the coefficients. This method allows the calculation of the confidence intervals and *p*-values of the coefficients by taking repeated samples from the dataset. Using 1000 bootstrap samples, the confidence intervals and *p*-values of the effect of each independent variable on each dependent variable were determined. The confidence intervals represent the 95% confidence intervals of the coefficients, and if the confidence interval of a coefficient does not include zero, this coefficient is considered statistically significant. The *p*-values indicate whether the effect of each independent variable on each dependent variable is significant. In all the statistical calculations, *p* ˂ 0.05 was accepted as significant.

## 3. Results

**Figure 1:**
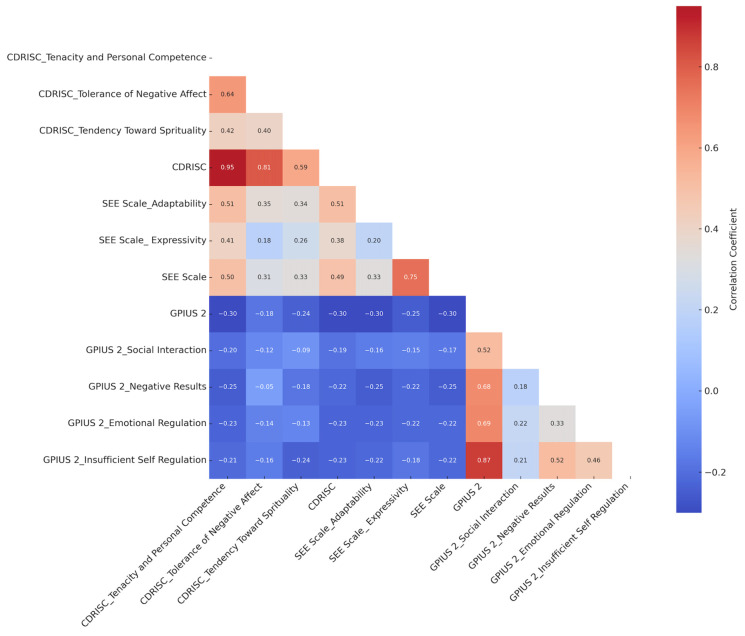
Correlation heatmap. CD-RISC: Connor–Davidson Resilience Scale; SEE Scale: Social–Emotional Expertise Scale; GPIUS 2: Generalized Problematic Internet Use Scale 2.

When the resilience and social–emotional expertise levels of university students were examined regarding problematic internet use, strong relationships were found between the CD-RISC sub-dimensions and the GPIUS 2 sub-dimensions, moderate positive relationships were found between the CD-RISC and the SEE Scale, and negative relationships were found between the GPIUS 2 sub-dimensions and the CD-RISC and SEE Scale. The relationships between resilience, social–emotional expertise and problematic internet use are shown in detail in Figure 1.

### 3.1. Elastic Net Regression Analysis Results

For the elastic net regression model, the alpha (α) was calculated as 0.0049 and the L1 ratio as 0.5. The mean square error of the model was found to be 3.768. The R^2^ value was calculated as 0.39, and it was determined that the model explained 39% of the variance in the dependent variable. These values show the significant effects of the resilience and social–emotional expertise sub-dimensions on problematic internet use. The detailed results of the regression analysis are presented in Table 1.

For the total score for resilience, β = −0.057 and *p* = 0.0015, which are statistically significant and show that problematic internet use among university students decreases with the increase in the total score for resilience. For the total score for social–emotional expertise, β = −0.048 and *p* = 0.0115, which are statistically significant and show that students with high social–emotional expertise tend to use the internet less problematically. Table 1 shows the effects of each independent variable on problematic internet use. While negative coefficients indicate that problematic internet use decreases with the increase in the relevant independent variable, positive coefficients indicate that problematic internet use increases with the increase in the relevant independent variable.

### 3.2. Partial Least Squares (PLS) Regression Analysis Results

According to the PLS results, the social interaction sub-dimension of problematic internet use among university students is positively affected by the tenacity dimension of resilience, and by the adaptability and expressivity sub-dimensions of social interaction. Negative results are negatively affected by the tolerance and spirituality sub-dimensions of resilience, and by the adaptability sub-dimension of social interaction. Table 2 shows the effect of each independent variable on each dependent variable.

The predictive performance of the PLS regression model was evaluated using the root mean squared error of prediction (RMSEP) scores. The RMSEP score for the social interaction sub-dimension was 1.083; for the negative consequences sub-dimension, it was 1.086; for the emotion regulation sub-dimension, it was 1.090; and for the self-regulation sub-dimension, it was 1.081. These scores indicate that the model’s predictions for each problematic internet use sub-dimension are reasonably accurate. Lower RMSEP scores and values closer to 1 indicate better model performance. The R-square (R^2^) value was calculated to calculate how much of the variance the model explains in the dependent variables. The R^2^ scores of the model were found to be 1.6% for the social interaction sub-dimension, 1.8% for the negative consequences sub-dimension, 1.3% for the emotion regulation sub-dimension, and 2.0% for the self-regulation sub-dimension. The findings suggest that the model only accounts for a portion of the variability in each subcategory of problematic internet use.

## 4. Discussion

The main goal of this research was to investigate how the levels of resilience and social–emotional skills among university students impact their tendency toward internet usage. This study specifically looked at how these factors influenced students’ internet usage habits. The analysis indicated that higher levels of resilience and social–emotional skills were associated with a decreased likelihood of students engaging in internet use. The elastic net regression model and PLS regression model were utilized to assess the impact of resilience and social–emotional expertise on aspects of internet use, uncovering significant connections between these variables.

There has been seen to be a relationship between the resilience of university students and their problematic internet usage. Resilience, which denotes an individual’s capacity to handle and adjust to circumstances (Dantzer et al., 2018), was found to be inversely related to internet use among students,. those with high resilience levels reported lower levels of internet use.

When the factors that constitute resilience are examined in detail, we see that tenacity, tolerance to negative events and spirituality play a role in the problematic internet use of university students. Tenacity can be described as the persistence displayed in reaching objectives. It refers to an individual’s capability to keep striving for their goals despite facing obstacles in their paths (Datu, 2021). Our research indicates that students with high levels of perseverance have a lower tendency toward problematic internet use. This implies that such individuals are less likely to turn to the internet as a means of escape when faced with challenges in achieving their goals. Hence, students with high levels of determination tend to use the internet responsibly and mindfully. Lopez-Fernandez et al., through their studies involving 15 countries, revealed that individuals with perseverance show lower levels of problematic internet use (Lopez-Fernandez et al., 2023). The quality of determination empowers university students to stay focused on their objectives. This may decrease the tendency to resort to the internet as an escape from distractions and obstacles they encounter.

Tolerance to negative events can be expressed as the ability to effectively manage and cope with stress and encountered problems (Armstrong et al., 2011). Our findings show that problematic internet use is less common among students with a high tolerance to negative events. This situation reveals that students with a high ability to cope with stress resort to excessive internet use. Lin et al. also emphasized that students with a low tolerance to negative situations use the internet unusually and use it as an escape route (Lin, 2022).

Spirituality can be defined as finding meaning and purpose in one’s life, and having a belief, and one can get support from these beliefs in difficult times (Womble et al., 2013). Our findings show that problematic internet use is lower in students with high levels of spirituality. The negative effect of spirituality on problematic internet use expresses the role of finding a purpose in one’s life through internet use behavior. Students with high levels of spirituality may be in a better position in terms of intrinsic satisfaction, and this may lead to a decrease in internet use for escape or satisfaction. A recent research study in Romania discovered that students who actively engage in activities tend to exhibit levels of problematic internet use. The study revealed that students who maintain a connection with God experience reduced negative effects associated with internet use (Goga & Bărcăcianu, 2019). It has also been suggested that engaging in rituals can enhance individuals’ internal satisfaction, leading to decreased reliance on the internet for escapism or fulfillment (Cai et al., 2023). This phenomenon can be attributed to the fact that spirituality injects significance into individuals’ lives and enhances their ability to navigate challenges. Moreover, the latest findings indicate that spiritual practices could assist university students in managing internet habits and underscore the value of support initiatives in this domain.

Our research indicates that the overall social and emotional expertise skills of university students have an impact on their use of the internet. These skills encompass aspects like empathy, social understanding, interpersonal skills and self-control (Coelho et al., 2015). Being adept at communicating with others and handling stress better can help diminish individuals’ excessive internet usage (Caplan, 2005). Studies highlight that enhancing emotional competencies plays a role in curbing excessive internet use among the youth (Chong et al., 2014; Gioia et al., 2021).

Adaptability, a sub-area within skills, refers to an individual’s capacity to adjust and respond effectively to new situations as circumstances evolve (Ay & Temel, 2021). This ability helps us navigate challenges and handle stress effectively. Our findings indicate that students demonstrating adaptability exhibit problematic internet use. Individuals who are adaptable, communicate effectively in relationships, and demonstrate flexibility in social situations are generally better equipped to cope with challenging environments. These qualities may help buffer the impact of stress and reduce the likelihood of excessive or problematic internet use. Previous studies have suggested that lower levels of agreeableness are linked to higher tendencies toward problematic internet use, which highlights the role of interpersonal difficulties in digital overuse (Alonso & Romero, 2020; Durak & Senol-Durak, 2014). Therefore, implementing strategies that aim to enhance university students’ adaptability and social–emotional skills can be an effective approach to managing such behaviors.

It has been observed that expressivity, one of the key components of social–emotional expertise, was positively associated with problematic internet use. However, this relationship was not statistically significant. Expressivity is used to define the capacity to express feelings and thoughts clearly and effectively (Ay & Temel, 2021). Individuals with high expressivity can generally participate in social interactions more easily and actively. However, according to our results, the insignificance of this dimension may indicate that expressivity alone is an insufficient criterion to prevent problematic internet use. Expressivity may need to be evaluated together with other social–emotional expertise skills. Sela et al. stated that low expression levels may cause problematic internet use in adolescents (Sela et al., 2020). However, studies examining the relationship between expressivity and problematic internet use are limited in the literature and more studies are needed in this area.

The findings of this study show that the sub-dimensions of the resilience and social–emotional expertise scales have different effects on the various sub-dimensions of problematic internet use. The sub-dimensions of problematic internet use were examined as social interaction, negative consequences, emotional regulation and inadequate self-regulation.

The social interaction domain is statistically significantly affected by the levels of tenacity, adaptability and expressivity. The positive effect of tenacity suggests that individuals with strong perseverance may actively use the internet to establish and maintain new social connections. Spirituality and tolerance to negative affect did not significantly predict social interaction, indicating that commitment to spiritual values or stress-coping skills may not be directly related to social engagement in digital environments. Students with high adaptability levels were also found to engage more frequently in online social interactions, which may reflect their success in navigating social relationships. However, previous studies have suggested that relying too heavily on online interactions, even among individuals with high adaptability, may reduce the quality or frequency of face-to-face relationships and eventually increase the risk of social isolation (Hajek & König, 2019). The positive association between expressivity and social interaction may be explained by the fact that individuals with strong expressive abilities can communicate more easily and confidently in digital spaces. Nevertheless, if this expressive tendency remains limited to online environments, it may reduce the opportunities for in-person connections and contribute to real-life social withdrawal.

In the negative outcomes domain, the tolerance to negative events, spirituality, and adaptability domains were found to be statistically significantly effective. Although tenacity hurt negative outcomes, this finding was not statistically significant. As tolerance to negative events increased, the frequency of negative outcomes resulting from internet use decreased. This may be associated with improved coping skills in stressful and negative situations. It was observed that students with high spirituality levels felt the effects of negative outcomes resulting from internet use less because individuals who are committed to spiritual values can use the internet more consciously and in a balanced way. Adaptability can play an important role in reducing the negative effects of internet use through the ability to quickly adapt to new or stressful situations (Dossi et al., 2022; Wang et al., 2023).

In the area of emotional regulation, the areas of tenacity, spirituality, adaptability, and expressivity were found to be statistically significantly effective. Tenacity has a positive effect on emotional regulation; this can increase the capacity of ambitious individuals to maintain their balance in difficult and stressful situations and manage their internet use. Spirituality can help individuals find emotional balance. Individuals with high levels of agreeableness can behave in a more balanced way on digital platforms, which can positively affect emotional regulation. Individuals with high levels of expressivity can overcome stressful and difficult situations even when exposed to them, thanks to their high expressivity (L. Chen et al., 2024; Wypych et al., 2018).

In the area of inadequate self-regulation, the areas of spirituality and expressivity were found to be statistically significantly effective. Although the level of grit hurts self-regulation, this result was not statistically significant. Tolerance to negative events does not affect self-regulation. Spirituality hurts self-regulation; individuals who are attached to their spiritual values may have difficulty self-regulating in digital environments. Expressivity has been found to hurt self-regulation; individuals with high expressivity may have difficulty controlling themselves in digital environments, which may lead to impulsivity and increased time spent online (C. Chen et al., 2021).

This study has some limitations. First, the data were collected in a single period, but problematic internet use may vary over time, and our results show the status of the students at the time they responded to the surveys. Second, the sample included only university students in Ankara; therefore, the generalizability of the findings is limited. Third, the data collection methods were based on self-reports from the participants, which poses a risk of social desirability bias. All the scales used in this study were self-report scales, meaning that the responses may be subjective. Future research should be conducted with larger and different demographic groups, and longitudinal designs should be used to verify the findings.

## 5. Conclusions

This study examined the effects of university students’ resilience and social–emotional expertise levels on problematic internet use. The research findings revealed that high levels of resilience and emotional skills were associated with a decrease in problematic internet use. Specifically, the tenacity, tolerance to negative events, and spiritual dimensions of resilience and the adaptability dimension of social–emotional expertise were identified as key factors influencing problematic internet use. These outcomes underscore the significance of implementing programs aimed at enhancing resilience and emotional skills among university students to foster better internet habits. By offering such initiatives, we can help young individuals cultivate positive online behaviors and combat issues like internet addiction effectively.

This study suggests that bolstering resilience and emotional skills can be a strategy for curbing problematic internet usage. Future research should delve deeper into how these factors can be nurtured and their differential impacts on groups. This study’s insights shed light on the role of resilience and emotional skills in mitigating problematic internet use among university students and encouraging responsible online practices. Educational and psychological support programs in universities may benefit from integrating components that enhance coping skills, emotional regulation, and interpersonal competencies. Supporting students in these areas may contribute to their overall well-being and promote healthier, more intentional use of digital technologies. Consequently, supporting people in leading healthy digital lives is crucial.

## Figures and Tables

**Table 1:** Results of the elastic net regression.

Independent Variables		β	SE	z	*p*
CD-RISC	Tenacity	−0.051	0.016	−3.188	0.0014 *
	Tolerance of negative affect	−0.043	0.017	−2.529	0.0114 *
	Spirituality	−0.033	0.015	−2.200	0.0278 *
	Total	−0.057	0.018	−3.167	0.0015 *
SEE Scale	Adaptability	−0.039	0.017	−2.294	0.0218 *
	Expressivity	0.031	0.018	1.722	0.0851
	Total	−0.048	0.019	−2.526	0.0115 *

CD-RISC: Connor–Davidson Resilience Scale; SEE Scale: Social–Emotional Expertise Scale; β: coefficient; SE: standard error; * *p* ˂ 0.05.

**Table 2:** Results of the partial least squares regression.

		GPIUS 2							
		Social Interaction		Negative Results		Emotion Regulation		Insufficient Self-Regulation	
		β	*p*	β	*p*	β	*p*	β	*p*
CD-RISC	Tenacity	0.10	0.04 *	−0.05	0.06	0.12	0.03 *	−0.08	0.07
	Tolerance	0.02	0.07	−0.07	0.05 *	0.04	0.06	−0.01	0.08
	Spirituality	0.03	0.06	−0.06	0.04 *	0.05	0.03 *	−0.04	0.05 *
SEE Scale	Adaptability	0.08	0.02 *	−0.09	0.05 *	0.07	0.03 *	−0.03	0.07
	Expressivity	0.05	0.03 *	−0.04	0.06	0.06	0.03 *	−0.07	0.04 *

CD-RISC: Connor–Davidson Resilience Scale; SEE Scale: Social–Emotional Expertise Scale; GPIUS 2: Generalized Problematic Internet Use Scale 2, β: coefficient; * *p* ˂ 0.05.

## Data Availability

Data will be made available on request.
